# Phenotypic Differentiation of Two Morphologically Similar Aflatoxin-Producing Fungi from West Africa

**DOI:** 10.3390/toxins12100656

**Published:** 2020-10-13

**Authors:** Pummi Singh, Hillary L. Mehl, Marc J. Orbach, Kenneth A. Callicott, Peter J. Cotty

**Affiliations:** 1 School of Plant Sciences, The University of Arizona, Tucson, AZ 85721, USA; pummi@illinois.edu (P.S.); orbachmj@arizona.edu (M.J.O.); 2 USDA-ARS, 416 W Congress St, First Floor, Tucson, AZ 85701, USA; ken.callicott@usda.gov; 3 College of Food Science and Engineering, Ocean University of China, Qingdao 266003, China

**Keywords:** aflatoxins, Aspergillus, *Aspergillus aflatoxiformans*, *Aspergillus minisclerotigenes*

## Abstract

Aflatoxins (AF) are hepatocarcinogenic metabolites produced by several *Aspergillus* species. Crop infection by these species results in aflatoxin contamination of cereals, nuts, and spices. Etiology of aflatoxin contamination is complicated by mixed infections of multiple species with similar morphology and aflatoxin profiles. The current study investigates variation in aflatoxin production between two morphologically similar species that co-exist in West Africa, *A. aflatoxiformans* and *A. minisclerotigenes*. Consistent distinctions in aflatoxin production during liquid fermentation were discovered between these species. The two species produced similar concentrations of AFB_1_ in defined media with either urea or ammonium as the sole nitrogen source. However, production of both AFB_1_ and AFG_1_ were inhibited (*p* < 0.001) for *A. aflatoxiformans* in a yeast extract medium with sucrose. Although production of AFG_1_ by both species was similar in urea, *A. minisclerotigenes* produced greater concentrations of AFG_1_ in ammonium (*p* = 0.039). Based on these differences, a reliable and convenient assay for differentiating the two species was designed. This assay will be useful for identifying specific etiologic agents of aflatoxin contamination episodes in West Africa and other regions where the two species are sympatric, especially when phylogenetic analyses based on multiple gene segments are not practical.

## 1. Introduction

Aflatoxins (AF) are potent carcinogenic metabolites produced by several *Aspergillus* species. Aflatoxin-producing aspergilli may infect a wide range of crops including cereals, groundnuts, cottonseed, tree nuts, and spices, and these infections frequently result in aflatoxin contamination of foods and feeds [1,2,3,4,5,6]. Aflatoxins are a serious health and economic threat worldwide [7]. Although *Aspergilli* produce four major aflatoxins, aflatoxin B_1_, B_2_, G_1_, and G_2_, aflatoxin B_1_ is the only mycotoxin listed as a human carcinogen by the International Agency for Research on Cancer [8]. Chronic exposure to aflatoxins results in reduced immunity, growth impairment, and hepatocellular carcinoma in humans and animals [9,10,11]. Consumption of food contaminated with high concentrations of aflatoxins has resulted in severe liver damage and rapid death [12]. Epidemics of acute lethal aflatoxicosis have occurred repeatedly in India, Kenya, and Tanzania [13,14,15,16]. Stringent enforcement of aflatoxin regulations in developed nations (e.g., total aflatoxins are regulated at 20 µg/kg in the United States) excludes contaminated foods, crops, and feeds from premium markets and may result in destruction of contaminated commodities. These regulatory interventions cause huge economic losses to processors, distributors and farmers [17]. 

Aflatoxin-producing *Aspergillus* species occur in highly diverse communities and this diversity can be resolved in a pinch of soil, harvested crops, and in the air [18,19]. Communities vary among fields, regions, and continents. This diversity complicates both the etiology and the management of contamination, with fungal community composition directly influencing severity of aflatoxin contamination of crops [18,19,20]. *Aspergillus flavus*, which produces only B aflatoxins, is the most frequently implicated causal agent of aflatoxin contamination [21]. This species is delineated into the L- and S-morphotypes, based on sclerotial size, that have been proposed to be adapted to different niches in the environment [22]. The L-morphotype produces few, relatively large sclerotia (>400 µm), abundant quantities of conidia, and variable concentrations of aflatoxins, ranging from the inability to produce aflatoxins (atoxigenic) to production of high aflatoxin concentrations [23,24]. The S-morphotype produces copious quantities of small sclerotia (<400 µm), sparse conidia, and consistently high concentrations of aflatoxins [23]. Atoxigenic L-morphotype isolates are active ingredients in several biological control products used to manage aflatoxin contamination of crops [25,26,27,28].

S-morphology aflatoxin producers can be important etiologic agents of contamination. However, individual S-morphology fungi vary in both the profile of aflatoxins produced and the conditions under which aflatoxin production occurs [29,30,31,32]. These physiologic differences can complicate both management and efforts to elucidate epidemiology. Over the past decade, several S-morphology aflatoxin-producing species have been delineated with molecular phylogenetics. Although these species are difficult to differentiate in the absence of DNA-based phylogenetics, differences among the species in distribution and frequencies across regions and continents is known [29,31]. Characterizing communities of fungi causing aflatoxin contamination to accurately ascribe etiology requires characterization of hundreds of isolates. In some regions where such work would be valuable, tools to apply phylogenetics in such a manner are not readily available. One such region is West Africa. Aflatoxin contamination of food and feed in West Africa has been attributed to S-morphology fungi with ability to produce both B and G aflatoxins (S_BG_), including *A. aflatoxiformans* and *A. minisclerotigenes* [33,34,35,36,37]. *Aspergillus aflatoxiformans*, previously reported as unnamed taxon S_BG_ or as *A. parvisclerotigenus*, has been frequently reported as an important cause of crop contamination in West Africa [33,37,38,39]. Although *A. minisclerotigenes* is also known from the region [37,40], it is not clear which species causes contamination events, with the exception of contamination of dried red chilies. Both *A. aflatoxiformans* and *A. minisclerotigenes* have an S-morphology and produce both B and G aflatoxins [36,37,41], rendering these species indistinguishable by either morphology or the particular aflatoxins produced. Consequently, *A. aflatoxiformans* and *A. minisclerotigenes* are distinguished with DNA-based phylogenetics utilizing multiple unlinked loci [37,41]. Both *A. aflatoxiformans* and *A. minisclerotigenes* can contaminate frequently consumed crops with high concentrations of aflatoxins [37]. Average aflatoxin-producing potential of fungal communities is a key determinant of aflatoxin contamination of crops. Both the frequency of infection and the aflatoxin-producing potential of a species indicate that species’ relative importance to the etiology of a contamination episode. Thus, accurate identification of causal agents of aflatoxin contamination is critical for predicting the risk of contamination and application of aflatoxin mitigation strategies [42].

Liquid fermentation has been used for over four decades to assess variation among *Aspergillus* species and strains in regulation of aflatoxin biosynthesis [29,30,43,44,45]. Effects of medium composition, pH, and aeration have been examined [44,45,46,47,48]. However, these early studies were performed without detailed knowledge of the phylogenetic divisions among the examined fungi. The current study utilized fermentation comparisons to evaluate variation in aflatoxin biosynthesis between two morphologically similar but phylogenetically distinct, S-morphology species from West Africa, *A. aflatoxiformans* and *A. minisclerotigenes*. Differences in aflatoxin production on common media were consistent with phylogenetic distinctions. These observations were refined into a simple liquid fermentation assay that can provide reliable differentiation of the two species when access to DNA-based methods for species identification is lacking. The results also suggest differences between the two taxa in the conditions under which aflatoxin contamination may occur. 

## 2. Results and Discussion

### 2.1. Aspergillus aflatoxiformans and A. minisclerotigenes Differ in Aflatoxin Production in Yeast Extract Sucrose (YES) Medium

Aflatoxin production by isolates of *A. aflatoxiformans* (*n* = 45) and *A. minisclerotigenes* (*n* = 44) was initially assessed by growth in YES medium (pH = 6.5) at 31 °C with agitation for seven days. This medium supported production of low concentrations of aflatoxins by *A. aflatoxiformans* (mean = 1.9 µg total aflatoxin g^-1^ mycelia; range = 0.02 to 5.65 µg/g), compared to *A. minisclerotigenes* (mean = 89 µg/g; range = 32 to 460 µg/g). Fourteen isolates from each species were re-evaluated in Adye and Mateles (A&M) medium supplemented with urea as the sole nitrogen source. In contrast to the above results, fungal isolates from both species produced high and similar concentrations of total aflatoxins (*p* = 0.64) in A&M medium with urea (Mean = 507 µg/g). Taken together, these results indicate that aflatoxin production by *A. aflatoxiformans* is much more inhibited than by *A. minisclerotigenes* in YES medium. This is a phenotypic distinction that differentiates these two morphologically similar species.

### 2.2. Aflatoxin Production by A. aflatoxiformans and A. minisclerotigenes in Liquid Fermentations

To further assess differential influences of medium composition on aflatoxin production by *A. aflatoxiformans* and *A. minisclerotigenes*, four isolates representative of each species from different geographic regions of Nigeria were assayed in three media, (i) YES, (ii) A&M supplemented with urea, and (iii) A&M containing ammonium with agitation. Each of these media support aflatoxin production by fungi with L- or S-morphology within section *Flavi* [29,44,45]. The two A&M media with different nitrogen sources have been utilized previously to assess aflatoxigenicity of isolates from soils and crops and to study the etiology of aflatoxin contamination [33,49,50,51]. *Aspergillus aflatoxiformans* and *A. minisclerotigenes* produced similar concentrations of aflatoxins B_1_ and G_1_ in the A&M medium with urea (*p* = 0.71 for AFB_1_ and *p* = 0.42 for AFG_1_), and individual isolates did not differ (Table 1; *p* > 0.05). Production of aflatoxins B_1_ and G_1_ in A&M medium containing ammonium differed among isolates (Table 1; *p* < 0.001). *Aspergillus minisclerotigenes* produced significantly greater concentrations of aflatoxin G_1_ than *A. aflatoxiformans* (*p* = 0.039) in A&M with ammonium, while the two species produced similar concentrations of aflatoxin B_1_ (Table 1; *p* = 0.71). Both species produced greater concentrations of aflatoxins in A&M with urea compared to A&M with ammonium. *Aspergillus aflatoxiformans* produced 7.5 times more aflatoxin B_1_ and 31 times more aflatoxin G_1_ on average in the medium with urea compared to that with ammonium (Table 1). *Aspergillus minisclerotigenes* produced 3.7 times more aflatoxin B_1_ and nearly 8 times more aflatoxin G_1_ in medium supplemented with urea versus ammonium (Table 1). These results are consistent with previous studies that reported increased production of aflatoxins B_1_ and G_1_ by African S_BG_ isolates in A&M medium containing urea compared to the medium with ammonium as the sole nitrogen source [29].

Production of both aflatoxins B_1_ and G_1_ by *A. aflatoxiformans* was significantly lower in YES medium (pH = 4.75) compared to that of *A. minisclerotigenes* (Table 1; *p* < 0.001)*,* as also observed during the initial evaluation of these fungi in YES medium (pH = 6.5). Isolates of *A. minisclerotigenes* produced at least 50 times more aflatoxin B_1_ and 25 times more aflatoxin G_1_ in YES medium compared to isolates of *A. aflatoxiformans* (Table 1). Since YES medium contained higher concentrations of sucrose (15%) compared to either of A&M medium (5%), the effect of sucrose concentration on aflatoxin production was tested in YES medium under shaking and stationary conditions. Total aflatoxin production was inhibited by *A. aflatoxiformans* in YES medium irrespective of sucrose concentration from 5–20% under shaking and stationary conditions at 31 °C (Table S1). Aflatoxin production, pH, and fungal biomass were higher when cultures were stationary versus shaking (Table S1).

### 2.3. pH Modification by A. aflatoxiformans and A. minisclerotigenes

All eight fungal isolates modified the pH of each medium during growth. Medium composition influenced the extent to which pH changed. At the end of fermentation, pH was in order of YES > A&M with urea > A&M with ammonium. Although the final pH of A&M medium with urea differed significantly among individual isolates of both species (Table 1; *p* < 0.001), the average final pH for *A. aflatoxiformans* did not differ from that of *A. minisclerotigenes* (Table 1; *p* = 0.83). The final pH of YES medium differed both among individual isolates (Table 1; *p* < 0.001) and between the two species (Table 1; *p* < 0.002). However, differences were minor, and the two species overlapped making final culture pH not useful for distinguishing the two species. It is noteworthy that aflatoxin production did not depend on the initial pH of YES medium because aflatoxin production in YES fermentations at either pH = 6.5 (initial aflatoxin screen assay) or at pH = 4.75 (Table 1) was much lower for *A. aflatoxiformans* than *A. minisclerotigenes* in YES irrespective of the pH. A&M medium with ammonium was the most acidic at the end of the fermentation, and the final pH did not differ among isolates (*p* = 0.498) or between species (*p* = 0.41). Similar pH modification was reported for the A&M media in previous studies [29,44]. The A&M medium containing ammonium was more acidic by the end of the fermentation compared to either A&M with urea or YES medium. Previously, influences of fungal growth on culture medium pH have been used to group aflatoxin producing fungi [23,31]. However, these groupings have not consistently reflected DNA-based phylogenetic relationships.

### 2.4. Growth of A. aflatoxiformans and A. minisclerotigenes in Liquid Fermentations

All isolates, irrespective of species, produced the highest biomass in YES medium, and mycelial mass did not differ among isolates (Table 1; *p* = 0.89) or between species (*p* = 0.33). Differences were detected in biomass production in A&M medium with ammonium among isolates (*p* < 0.01) but not between species (*p* = 0.28). Fungal growth was significantly different among isolates and between species in A&M medium containing urea (*p* < 0.001); *A. minisclerotigenes* produced greater biomass in this medium (Table 1; *p* < 0.001). Concentrations of aflatoxins B_1_ and G_1_ produced in each medium was independent of fungal growth and biomass production. Although *A. aflatoxiformans* produced the least concentration of aflatoxins in YES medium, it produced the greatest mycelial mass in this medium. Its mycelial mass was comparable to that of *A. minisclerotigenes* indicating that decreased production of aflatoxins by *A. aflatoxiformans* was not due to influences on growth (Table 1). Nutrient influences on aflatoxin biosynthesis [30] may have resulted in the observed differences. 

### 2.5. Susceptibility of Maize to Aflatoxin Contamination by A. aflatoxiformans and A. minisclerotigenes

In order to assess the aflatoxin-producing potential of *A. aflatoxiformans* and *A. minisclerotigenes* under different environmental conditions, aflatoxin production by the two species was further evaluated on maize at 25 °C, 30 °C, 35 °C, and 40 °C (Table 2). Maize is an important staple in Nigeria, and average annual production exceeds 10 million metric tons per year [52]. It is estimated that Nigerians may be exposed to ~5.0 mg aflatoxin person^-1^ year^-1^ through maize consumption [53]. Temperatures were chosen based on climate data from maize growing regions in Nigeria [54,55] and temperatures typically encountered during storage. Overall, aflatoxin production by members of both species was high at 25 °C, 30 °C and 35 °C, and *A. aflatoxiformans* produced significantly greater concentrations of total aflatoxins compared to *A. minisclerotigenes* at each of these temperatures (Table 2; *p* < 0.001). Furthermore, isolates differed in aflatoxin-producing potential at 25 °C, 30 °C and 35 °C (*p* < 0.001), and some *A. minisclerotigenes* isolates produced concentrations of aflatoxins similar to those of *A. aflatoxiformans* (Table 2). The greatest concentrations of aflatoxins were observed at 30 °C followed by 35 °C and 25 °C for each species. Notably, although fungal isolates produced the least aflatoxin at 25 °C (Range: 13.0–154 µg/g), the observed concentrations are still unacceptable and dangerous for human and animal consumption. Neither *A. aflatoxiformans* nor *A. minisclerotigenes* produced detectable concentrations of aflatoxins at 40 °C (LOD = 0.42 µg/g of maize grain). However, *A. aflatoxiformans* produced on average 5.6 and 11.7 fold more aflatoxins than *A. minisclerotigenes* at 30 °C and 35 °C, respectively. These temperatures are typical of maize production areas in Nigeria. Due to the ability of *A. aflatoxiformans* to produce greater concentrations of aflatoxins in maize compared to *A. minisclerotigenes*, even if the two species are present at similar frequencies, *A. aflatoxiformans* may be considered the more important causal agent of aflatoxin contamination in Nigeria. Nevertheless, the aflatoxin-producing potential of *A. minisclerotigenes* is sufficient to render it potentially dangerous in terms of crop contamination. The results on YES medium (Table 1) suggest that on crops with different nutrient compositions, *A. minisclerotigenes* may be the more important causal agent. It is therefore important to develop methods/techniques that can differentiate etiologic agents of crop contamination when mixed infections by several fungal species occur.

### 2.6. Assay to Differentiate A. aflatoxiformans and A. minisclerotigenes

We tested the utility of YES medium to reliably differentiate *A. aflatoxiformans* from *A. minisclerotigenes* by evaluating total aflatoxin production of 11 representative isolates of each species grown in YES medium for 3 days at 31°C with and without agitation. Different sets of isolates were used in this assay from those used in the previous experiment to validate the ability of the observed aflatoxin production phenotypes to differentiate the two species. Reference isolates of both species were included (NRRL A-11612 of *A. aflatoxiformans* and NRRL A-11611 of *A. minisclerotigenes*). To extend the utility of the assay to laboratories where incubators with shakers may not be available, cultures were grown under stationary conditions as well as with shaking incubation. Results were similar to those previously observed, with isolates of *A. minisclerotigenes* producing greater concentrations of total aflatoxins in YES medium under either shaking (17.0 to 300 µg/g) or stationary (25.4 to 1,052 µg/g) conditions (Table 3). These same isolates were further tested for aflatoxin production in A&M medium with urea as the sole nitrogen source, and the ratio of total aflatoxins in the urea medium to total aflatoxins in YES medium was calculated for each isolate. Overall, aflatoxin concentrations were greater for all isolates when grown under stationary versus shaking conditions irrespective of species and medium (Table 3). Isolates of *A. aflatoxiformans* produced at least 122 and 124 times more total aflatoxins in A&M medium with urea versus YES medium, with and without agitation, respectively. However, ratios of aflatoxin production by *A. minisclerotigenes* in A&M medium with urea versus YES medium were in the range of 0.86–13.3 with agitation and 0.23–11.9 when stationary. Based on these results, fungal isolates can be identified as *A. aflatoxiformans* when ratios of aflatoxin concentrations in A&M medium with urea versus YES medium are greater than 80, and as *A. minisclerotigenes* when ratios are less than 80, with or without agitation (Table 1 and Table 3). This ratio cutoff point was selected to allow for any potential outlier isolates from each species based on results in Table 1 and Table 3.

## 3. Conclusions

*Aspergillus aflatoxiformans* and *A. minisclerotigenes* have indistinguishable morphologies, overlapping secondary metabolite profiles, and ability to produce high concentrations of aflatoxins in both synthetic media and crops [29,37,41]. Aflatoxin contamination of crops in West Africa has often been attributed to *A. aflatoxiformans* due to the aflatoxin-producing ability of this species and its frequency of occurrence in agricultural soils and crops [33,34,35]. In contrast, only a single isolate of *A. minisclerotigenes* was reported from West Africa [31,41] until recently when *A. minisclerotigenes* was reported in Nigerian chilies at high frequencies (8% of all *Aspergillus* section *Flavi* isolates), confirming the co-occurrence of *A. aflatoxiformans* and *A. minisclerotigenes* in West Africa [37]. *Aspergillus minisclerotigenes* was first described a decade ago, and it was reported that only DNA sequences could separate *A. minisclerotigenes* and *A. aflatoxiformans* (referred to as *A. parvisclerotigenus*) [41]. Both *A. minisclerotigenes* and *A. aflatoxiformans* can contaminate widely consumed crops such as maize, groundnuts, and chilies with unacceptable concentrations of aflatoxins [37]. Furthermore, both species produced toxic concentrations of aflatoxins at 25 °C, 30 °C, and 35 °C in maize (Table 3), indicating hazardous potential at temperatures that are prevalent both pre- and post-harvest. More than 99% of the human population in several areas of West Africa suffers chronic exposure to aflatoxins due to consumption of contaminated cereals [53,56]. This creates a clear need for reliable detection and identification of etiologic agents of aflatoxin contamination of crops in this region. Attribution of etiologies is complicated by sympatric species, such as *A. minisclerotigenes* and *A. aflatoxiformans,* that share morphological characteristics. Such attribution is especially challenging where DNA-based technologies and the necessary technical expertise may not be easily available or accessible. The microbiological assay developed during the current study is based on differential aflatoxin production in YES medium. This assay utilizes simple liquid fermentation to differentiate *A. aflatoxiformans* from *A. minisclerotigenes* within 72 h.

## 4. Materials and Methods

### 4.1. Fungal Isolates and Inoculum Preparation

Isolates of *A. aflatoxiformans* (*n* = 45) and *A. minisclerotigenes* (*n* = 43) previously recovered from dried red chilies produced in Nigeria [37] were included in this study. The fungal inoculum was prepared for each isolate as described previously [44,57]. Briefly, conidial suspensions from water vials were centrally seeded onto 5/2 agar (5% V8 juice, 2% agar, pH = 6.0) and isolates were grown in the dark for 5–7 days at 31 °C. Conidia were swabbed with sterile cotton swabs and transferred into glass vials with Teflon septa containing 10 mL sterile ultrapure water. Conidial concentrations were estimated with a turbidity versus colony forming unit curve [58], and the final concentration of each suspension was adjusted to 10^6^ conidia/mL.

### 4.2. Liquid Fermentation Assays and Assessment of Aflatoxin Production 

Three different liquid media were used to evaluate aflatoxin production by fungal isolates: Yeast extract and sucrose medium [45] and Adye and Mateles (A&M) medium [43] amended with either 22.5 mM ammonium sulfate ((NH_4_)_2_SO_4_) or 22.5 mM urea as the sole nitrogen source. The exact compositions of liquid media were previously reported [44]. Urea was filter sterilized and added aseptically to autoclaved medium, while ammonium sulfate was added prior to autoclaving the medium [44].

Aflatoxin production by *A. aflatoxiformans* (*n* = 45) and *A. minisclerotigenes* (*n* = 43) was initially evaluated in YES medium (pH = 6.5). Erlenmeyer flasks containing 70 mL of the medium were aseptically inoculated with conidial suspensions (10^6^ conidia/mL of the suspension; 100µL/flask) and incubated in the dark for 7 days at 31 °C with agitation. At the end of the fermentation period, aflatoxins were extracted by the addition of 70 mL acetone to each 70 mL fermentation. After acetone addition, cultures were allowed to sit in the dark for an hour with periodic swirling to increase mixing. Acetone extracts were separated from the fungal mycelia by filtering the contents of the fermentation flasks through Whatman no.4 filter paper using vacuum filtration. Mycelia were dried in a forced air oven (40 °C, 48 h) and weighed to determine the total biomass (dry weight). Acetone extracts (4 µL) were directly spotted onto thin layer chromatography (TLC) plates (Silica gel 60, EMD, Darmstadt, Germany) and separated with ether:methanol:water (96:3:1) adjacent to 4 µL of aflatoxin standard containing 1µg B_1_, 1µg G_1_, 0.3 µg B_2_, and 0.3 µg G_2_ per mL of benzene:acetonitrile (98:2) (Aflatoxin Mix Kit-M Supelco, Bellefonte, PA). Aflatoxins were measured on TLC plates by scanning fluorescence densitometry under 365 nm UV light (TLC Scanner 3, Camag Scientific Inc., Wilmington, NC, USA). If aflatoxin was not detected, 12 µL of the extract was spotted onto TLC plates and analyzed as described above. Aflatoxin quantities (total µg) on TLC plates were determined by comparing the area under the peaks generated by each sample to the area under the peaks for the corresponding aflatoxin standard (aflatoxin B_1_, B_2_, G_1_, and G_2_) generated by the TLC scanner. Total µg of aflatoxins detected on the TLC plate were divided by the proportion of the total volume of the original extract spotted on the TLC plate to calculate the µg aflatoxin per fermentation. Total aflatoxin per fermentation was divided by the dry weight of the mycelia to determine the µg aflatoxin produced per µg mycelia in each fermentation. If aflatoxin was not detected from 12 µL, the extract was partitioned twice with dichloromethane and concentrated prior to quantification as previously described [33]. Total µL of aflatoxins were estimated as mentioned above from a concentrated extract volume of 4 mL.

Based on results from initial screening of isolates in YES medium, four representative isolates each of *A. aflatoxiformans* and *A. minisclerotigenes* were chosen for the evaluation of aflatoxin production in YES, A&M with ammonium sulfate, and A&M with urea media. Fungal isolates NRRL A-11612 and NRRL A-11611 from Nigerian groundnuts [31] were used as reference isolates of *A. aflatoxiformans* and *A. minisclerotigenes,* respectively. The remaining six isolates were chosen to represent isolates recovered from chilies sampled from different locations in Nigeria. Inoculum for each of the eight isolates was prepared as described above. Isolates were inoculated aseptically into each of the three media to a final concentration of 10^6^ conidia/mL. Treatments were replicated four times. All media were adjusted to pH = 4.75 before autoclaving. Fermentations were carried out for 7 days in the dark at 31 °C after which pH was measured, and the experiment was terminated by addition of 70 mL acetone (50% acetone vol/vol). Aflatoxins were extracted, concentrated, and quantified as described above.

In order to test the effect of sucrose concentration in YES medium on aflatoxin production by members of *A. aflatoxiformans* and *A. minisclerotigenes,* isolates were inoculated into YES medium (pH = 4.75) containing 5%, 10%, 15%, and 20% sucrose. Isolates were replicated three times and incubated with and without agitation for 3 days at 31 °C in the dark. Total aflatoxins, mycelial mass, and pH were measured after incubation as described above.

A microbiological assay utilizing YES and A&M with urea media was designed to differentiate *A. aflatoxiformans* and *A. minisclerotigenes*. Eleven isolates each of *A. aflatoxiformans* and *A. minisclerotigenes* were evaluated for aflatoxin production under shaking and stationary conditions for 3 days at 31°C. Fungal isolates were selected such that isolates were representative of location and year of sampling. Aflatoxin concentrations, biomass production, and pH were estimated at the end of the incubation period. Ratios of aflatoxin concentrations produced in A&M medium with urea to that in YES were calculated.

### 4.3. Aflatoxin Production in Maize Grain

Fungal isolates evaluated for aflatoxin production in liquid fermentations (Table 1) were also assessed for aflatoxin production in maize (*Zea mays* L.) (Table 2). Healthy, undamaged maize kernels adjusted to 25% moisture were autoclaved in Erlenmeyer flasks (10 g per flask) for 20 min at 121 °C. Aflatoxin production on autoclaved maize is a good predictor of aflatoxin production in viable maize kernels [44]. Maize was inoculated with 100 µl of conidial suspensions (10^6^ conidia/mL), adjusted to 30% moisture, and incubated for 7 days at 25 °C, 30 °C, 35 °C and 40 °C in the dark. Each treatment was replicated three times. At the end of the incubation period, maize-fungal cultures were ground in 50 mL, 85% acetone, in a Blender (Seven-Speed laboratory blender, Waring Laboratory, Torrington, CT, USA) at full speed for 30 s. Aflatoxins were extracted and quantified as previously reported [59].

### 4.4. Data Analysis

Aflatoxin concentrations and fungal biomass were expressed in µg/g and g of dried mycelial weight, respectively. Aflatoxins produced by individual isolates, pH at the end of incubation, and fungal biomass were analyzed using Analysis of Variance as implemented in JMP 11.1.1 (SAS Institute, Cary, NC, USA, 2013). Means were separated using Tukey’s HSD test (*p* = 0.05). Differences in mean aflatoxin concentrations, pH, and mycelial mass between species were compared using Student’s t-test (*p* = 0.05). Data were tested for normality and, if required, log transformed before analysis. True means are presented for clarity. All experiments were conducted with a randomized complete block design.

## Figures and Tables

**Table 1:** Production of aflatoxins B_1_ and G_1_ by *A. aflatoxiformans* and *A. minisclerotigenes* in liquid fermentations containing YES or Adye and Mateles (A&M) medium supplemented with either urea or ammonium.

Species^#^	Isolate	Aflatoxin B_1_ (µg/g)			Aflatoxin G_1_ (µg/g)			Final pH			Mycelia (g)		
		YES	Urea	NH_4_	YES	Urea	NH_4_	YES	Urea	NH_4_	YES	Urea	NH_4_
AA	A-11612	1.50^C^	1265	52.9^BC^	1.81^C^	803	9.9^BCD^	4.71^B^	3.74^ABC^	2.22	0.96	0.66^CD^	0.74^AB^
	CHL568	2.07^C^	710	132^ABC^	1.13^C^	357	15.1^BCD^	4.22^C^	3.75^ABC^	2.23	0.93	0.74^BC^	0.83^A^
	CHL740	1.64^C^	383	119^AB^	0.67^C^	280	21.0^BC^	4.34^C^	3.62^BCD^	2.23	0.81	0.63^D^	0.67^B^
	CHL877	1.85^C^	496	76.2^BC^	1.06^C^	104	3.32^D^	4.43^C^	3.63^BCD^	2.22	1.02	0.70^BCD^	0.74^AB^
AM	A-11611	103^B^	292	104^AB^	46.7^AB^	129	33.3^AB^	5.05^A^	3.59^CD^	2.21	0.76	0.77^AB^	0.71^B^
	CHL603	108^B^	288	24.8^C^	48.7^AB^	370	5.78^CD^	4.74^B^	3.78^AB^	2.19	0.86	0.75^BC^	0.76^AB^
	CHL707	95.2^B^	250	27.3^BC^	45.3^B^	344	7.91^BCD^	4.74^B^	3.83^A^	2.18	0.87	0.78^AB^	0.73^AB^
	CHL845	623^A^	951	327^A^	363^A^	469	121^A^	4.34^C^	3.51^D^	2.25	0.85	0.85^A^	0.69^B^
AA Average		**1.77^y^**	**713**	**94.8**	**1.17^y^**	**386**	**12.3^y^**	**4.43^y^**	**3.69**	**2.22**	**0.93**	**0.69^y^**	**0.75**
AM Average		**232^x^**	**445**	**121**	**126^x^**	**328**	**41.9^x^**	**4.72^x^**	**3.68**	**2.21**	**0.83**	**0.79^x^**	**0.72**

^#^ Species assignment; AA- A. aflatoxiformans, and AM- A. minisclerotigenes. Concentrations of aflatoxins B_1_, G_1_, final pH of the liquid medium, and mycelial mass produced by individual isolates were compared by column for each medium tested. Values are means of four replicates. Means followed by same upper-case letters do not differ (Tukey’s HSD; *p* > 0.05); Differences in average aflatoxin B_1_ and G_1_ concentrations, final pH, and mycelia produced by *A. aflatoxiformans* and *A. minisclerotigenes* were compared for each medium tested. The mycelia were captured on #4 Whatman filter paper during filtration of the acetone extracted culture. Dry weights were determined after drying in a forced air oven for 48 h at 40ºC. Means followed by different lower-case letters in bold differ (Student’s t-test, *p* < 0.01). Values within a column lacking a letter do not differ (*p* > 0.05).

**Table 2:** Aflatoxin production by *A. aflatoxiformans* and *A. minisclerotigenes* in maize at various temperatures.

Species	Isolate	Total Aflatoxin (µg/g)		
		25 °C	30 °C	35 °C
*A. aflatoxiformans*	A-11612	128a	573abc	283a
	CHL568	154a	655ab	544a
	CHL740	146a	612ab	541a
	CHL877	84a	756a	584a
*A. minisclerotigenes*	A-11611	14.4b	34.6e	17.9c
	CHL707	13.0b	67.2de	27.9c
	CHL845	26.9b	165cd	90.0b
	CHL603	88.8a	196bcd	30.7c
Average *A. aflatoxiformans*		**128A**	**649A**	**488A**
Average *A. minisclerotigenes*		**35.8B**	**116B**	**41.6B**

Concentrations of total aflatoxins produced by individual isolates were compared by column for each temperature. Values are means of three replicates. Means followed by different lower-case letters differ (Tukey’s HSD; *p* < 0.001). Differences in aflatoxin concentrations produced by *A. aflatoxiformans* and *A. minisclerotigenes* are indicated by bold upper-case letters (Student’s t-test, *p* < 0.001).

**Table 3:** Ratios of total aflatoxins produced in the A&M medium with urea to that in YES medium with agitation and under stationary conditions for *Aspergillus minisclerotigenes* (AM) and *A. aflatoxiformans* (AA).

Species	Isolate	With Agitation			Without Agitation		
		Total AF (µg/g)		Ratio	Total AF (µg/g)		Ratio
		Urea	YES		Urea	YES	
AA	A-11612	3929	1.14	3447	2423	3.99	608
	CHL514	831	1.75	475	1556	8.15	191
	CHL562	749	1.42	527	2636	5.42	486
	CHL596	907	1.38	659	2237	2.99	748
	CHL633	278	1.00	278	188	0.26	732
	CHL675	2634	2.40	1099	3382	27.3	124
	CHL731	1600	1.46	1100	1563	0.68	2296
	CHL812	1554	2.36	658	1710	4.19	408
	CHL819	490	0.63	777	1783	12.6	142
	CHL856	923	0.79	1170	937	3.32	282
	CHL878	2008	16.5	122	8902	9.09	980
AM	A-11611	93.4	17.0	5.48	59.3	87.2	0.68
	CHL583	581	76.5	7.59	811	74.5	10.9
	CHL621	440	33.2	13.3	453	37.9	12.0
	CHL636	147	19.6	7.47	226	25.4	8.89
	CHL644	208	20.6	10.1	152	31.7	4.78
	CHL661	504	67.0	7.52	697	100	6.96
	CHL674	260	300	0.86	347	1052	0.33
	CHL690	218	73.9	2.94	81.0	346	0.23
	CHL799	139	30.3	4.58	190	98.5	1.93
	CHL895	98.3	83.6	1.18	231	76.2	3.03
	CHL947	45.2	46.8	0.97	71.1	56.4	1.26

Total aflatoxins are expressed as µg/g mycelial weight. The mycelia were captured on #4 Whatman filter paper during filtration of the acetone extracted culture. Dry weights were determined after drying in a forced air oven for 48 h at 40 °C.

## Supplementary Materials

The following are available online at https://www.mdpi.com/2072-6651/12/10/656/s1, Table S1: Production of total aflatoxins by *A. aflatoxiformans* (AA) and *A. minisclerotigenes* (AM) in the Yeast Extract and Sucrose (YES) medium with different sucrose concentrations during fermentation.
